# Polyphenols are responsible for the proapoptotic properties of pomegranate juice on leukemia cell lines

**DOI:** 10.1002/fsn3.26

**Published:** 2013-02-20

**Authors:** Haytham Dahlawi, Nicola Jordan-Mahy, Malcolm Clench, Gordon J McDougall, Christine Lyn Maitre

**Affiliations:** 1 Biomedical Research Centre, Sheffield Hallam UniversitySheffield, U.K; 2 Environmental and Biochemical Sciences, The James Hutton InstituteInvergowrie, Dundee, U.K

**Keywords:** Apoptosis, leukemia, polyphenols, pomegranate

## Abstract

Pomegranates have shown great promise as anti-cancer agents in a number of cancers including clinical trials in prostate cancer. We have previously shown pomegranate juice (PGJ) induced apoptosis and preferentially alters the cell cycle in leukemia cell lines compared with nontumor control cells. However, the agents responsible have not yet been fully elucidated. Treatment of four leukemia cell lines with five fractions obtained from PGJ by solid phase extraction demonstrated that only the acetonitrile fractions decreased adenosine triphosphate (ATP) levels in all leukemia cell lines. Acetonitrile fractions also significantly activated caspase-3 and induced nuclear morphology characteristic of apoptosis. S phase arrest was induced by acetonitrile fractions which matched S phase arrest seen previously following whole PGJ treatments. The acetonitrile fractions contained higher phenol content than whole PGJ whereas only low levels of phenols were seen in any other fraction. Liquid chromatography mass spectrometry (LC–MS) analysis demonstrated that acetonitrile fractions were enriched in ellagitannins, ellagic acid, and hydroxycinnamic acid derivatives but depleted in anthocyanins. Individual treatments with identified compounds demonstrated that the ellagitannin: punicalagin was the most active and mimicked the responses seen following acetonitrile fraction treatment. Bioactive components within pomegranate were confined to the acetonitrile fraction of PGJ. The enrichment in ellagitannins and hydroxycinnamic acids suggest these may provide the majority of the bioactivities of PGJ. Individual treatments with compounds identified demonstrated that the ellagitannin: punicalagin was the most active agent, highlighting this compound as a key bioactive agent in PGJ.

## Introduction

Despite improvement in early detection and advances in treatments, leukemia continues to be a major cause of morbidity and mortality worldwide (Cancer Research U.K. 2011). Treatment options remain limited and are fraught with adverse side effects (Fielding et al. 2012; Richardson et al. 2012; Shanshal and Haddad 2012; Suttorp et al. 2012). Thus, studies into the use of nontoxic dietary agents are rapidly gaining ground and many natural agents are currently under investigation as potential chemo-preventive as well as chemotherapeutic agents including investigations in a number of clinical trials (Khan et al. 2008; Syed et al. 2008; Paller et al. 2012).

The fruit of the pomegranate tree (*Punica granatum*) has shown great promise as an anti-cancer agent in lung (Khan et al. 2007), prostate (Paller et al. 2012), skin (Afaq et al. 2009), colon (Adams et al. 2006), and breast cancer (Kim et al. 2002), which has been taken into phase II clinical trials in prostate cancer (Adhami et al. 2009; Paller et al. 2012). We have previously shown that crude extracts of pomegranate juice (PGJ) induce apoptosis and inhibit cell cycle in a number of leukemia cell lines, which demonstrated greater sensitivity than nontumor control cells (Dahlawi et al. 2012).

However, to date, the compounds responsible for the anti-leukemic properties of pomegranate remain unknown. PGJ contains a number of potential active compounds including organic acids, vitamins, sugars, and phenolic components. The phenolic components include phenolic acids: principally, hydroxybenzoic acids (such as gallic acid and ellagic acid) (Amakura et al. 2000); hydroxycinnamic acids (such as caffeic acid and chlorogenic acid) (Elfalleh et al. 2011); anthocyanins, including glycosylated forms of cyanidin, delphinidin, and pelargonidin (Fanali et al. 2011; Krueger 2012); and gallotannins and ellagitannins (Amakura et al. 2000). In addition, PGJ contains glucose, fructose, water, and organic acids (including ascorbic and citric acid) (Krueger 2012). However, the concentration and the contents of these compounds vary due to growing region, climate, cultivation practice, and storage conditions (Pande and Akoh 2009; Elfalleh et al. 2011; Legua et al. 2012).

Here, we used solid phase extraction (SPE) to fractionate PGJ and determine which fractions induced apoptosis and cell cycle arrest using two lymphoid and two myeloid cell lines, which we previously demonstrated were sensitive to crude pomegranate extracts (Dahlawi et al. 2012). We assessed the polyphenol composition of the fractions by liquid chromatography mass spectrometry (LC–MS) to identify active compounds within fractions of PGJ, and treated cells individually with pure compounds identified to investigate potential agents.

## Materials and Methods

### Sample preparation and fraction separation from PGJE

SPE using Strata C18E GIGA tubes (Phenomenex, Hartsfield, U.K.) was used to separate fractions from pomegranate juice extract (PGJE). First, tubes were pre-equilibrated by washing with 50 mL of acetonitrile then 50 mL of 0.1% formic acid/ultra pure water (v/v) (Sigma, Poole, U.K.). PGJE derived from fresh pomegranates (Sainsbury's U.K.) prepared as previous (Dahlawi et al. 2012) were sterile filtered through 0.22 μm syringe filter (Invitrogen, Paisley, U.K.) and diluted 1:1 with sterile distilled water, then 50 mL of this diluted juice was added to the SPE unit. The unbound compounds which passed directly through the column were collected (fraction A: unbound fraction). Then, 50 mL each of ultra pure water, acetonitrile, acetone, and ethyl acetate were added to the tube to collect water (fraction B), acetonitrile (fraction C), acetone (fraction D), and ethyl acetate (fraction E) fractions, respectively, as described previously (Zaini et al. 2012). Each fraction was dried using freeze drying (MODULYOD-230 freeze dryer, Thermo, U.K.) at −30 to −80°C for fractions A and B or dried under a nitrogen stream at room temperature for fractions C, D, and E. The water, unbound, and acetonitrile fractions were dissolved in distilled water; the acetone fraction in methanol; and ethyl acetate fraction in DMSO to the required dilution (Sigma, Poole, U.K.).

### Treatments

The dried weight (DW) of each SPE fraction was recorded and added to give the total weight of all dried fractions. Dried fractions were then resuspended in appropriate solvents to give a concentration (mg DW/mL) equal to that of the 6.25% dilution of whole PGJE used previously (Dahlawi et al. 2012). Cells (0.5 × 10^6^ per well) were then treated with 1, 2, and 4 μL of each fraction solution, which was equivalent to the concentration in 6.25%, 12.5%, and 25% PGJE (as studied previously (Dahlawi et al. 2012)) for 48 and 72 h.

### Cell lines and culture

Four leukemia cell lines were obtained from the American Type Culture Collection (ATCC; Middlesex, U.K.), which our previous study demonstrated differing sensitivity to PGJE (Dahlawi et al. 2012). Two lymphoid cell lines: CCRF-CEM (acute lymphoblastic leukemia) (ATCC: CCL-119), and MOLT-3 (acute lymphoblastic leukemia patient released following chemotherapy) (ATCC: CRL-1552), and two myeloid cell lines: HL-60 (Human promyelocytic leukemia) (ATCC: CCL-240) and THP-1 (acute monocytic leukemia) (ATCC: TIB-202). Cell lines were maintained and cultured in RPMI 1640 medium (Invitrogen, Paisley, U.K.) supplemented with 10% (v/v) fetal bovine serum, 1.5 mmol/L l-glutamine, and 100 μg/mL penicillin/streptomycin (complete media) in a humidified atmosphere of 5% CO_2_ at 37°C.

Cells were seeded in 12-well plates (Fisher Scientific, Loughborough, U.K.) at a cell density of 0.5 × 10^6^ cells per well and treated individually with the five fractions (unbound [i.e., compounds which passed directly through the column] and fractions eluted with water, acetonitrile, acetone, and ethyl acetate fractions) at equivalent compound weights seen in 0%, 6.25%, 12.5%, and 25% whole PGJE, all treatments were performed in triplicate. Following treatment, cells were investigated for induction of apoptosis and inhibition of cell proliferation following 24, 48, and 72 h.

### Cell viability

The effect of SPE fractions (unbound, water, acetonitrile, acetone, and ethyl acetate) on the viability of cells was determined by the Cell Titer-Glo® Luminescent Cell Viability Assay (Promega, Southampton, U.K.) which measures total adenosine triphosphate (ATP) present, indicating the number of metabolically active cells. Cells were seeded at 25 × 10^4^ cells in 100 μL of complete media and treated with each fraction at a concentration equivalent to that seen in 0%, 6.25%, 12.5%, and 25% whole PGJE in white 96-well plate (Fisher Scientific) for 72 h. Control wells were prepared which contained only medium or treatments without cells to determine background luminescence. Following 48 h incubation, the plate and its contents was equilibrated at room temperature for 30 min. Then, 100 μL of Cell Titer-Glo® Reagent was added to each well, mixed well for 2 min on an orbital shaker at 400 rpm, and then incubated at room temperature for 10 min prior to luminescence detection using Wallac 1420 luminescence detector (PerkinElmer, Cambridge, U.K.).

### Caspase-3 activity

NucView™ 488 Caspase-3 substrate (Cambridge Bioscience, Cambridge, U.K.) was used for detection of apoptosis. Cells were plated in 12-well plates (Fisher Scientific) and then treated with PGJE fractions (unbound, water, acetonitrile (ACN), acetone, and ethyl acetate) at concentrations equivalent to their component concentrations seen in 0–25% whole PGJE for 48 h. Following incubation, 200 μL of cell suspension was added to flow cytometry tubes and 5 μL of NucView™ 488 Caspase-3 substrate was added directly into the cell suspension and mixed well. Cells were incubated for 20 min at room temperature protected from light. Following incubation, 300 μL of Dulbecco's phosphate-buffered saline (DPBS) (Invitrogen, Paisley, U.K.) was added and samples were analyzed directly on a FACS Calibur Cytometer (Becton- Dickinson, U.K.). Data were recorded from 10,000 cells per sample and analyzed using FlowJo software (Tree Star, Ashland, OR).

### Annexin V/PI FITC flow cytometry assay

Annexin V/PI fluorescein isothiocyanate (FITC) stains were used to detect apoptosis based on flow cytometry as described previously (Dahlawi et al. 2012). Data were recorded from 10,000 cells per sample and analyzed using FlowJo software (Tree Star).

### Hoechst 33258 and propidium iodide staining

Hoechst 33258 and propidium iodide (PI) staining was used to observe the apoptotic/necrotic morphology of CCRF-CEM, MOLT-3, HL-60, and THP-1 cells. In brief, 0.25 × 10^5^ cells/mL were seeded into 12-well plates and incubated for 24 h following treatment with acetonitrile fractions at concentrations equivalent to component concentrations seen in 0–25% whole PGJE. Then, 1 μL of PI (50 μg/mL) and 5 μL of Hoechst 33258 (10 mg/mL) stains were added to each well and incubated at 37°C for 15 min. Cells were investigated using Olympus inverted fluorescence microscope at excitation wavelength 350 nm and 488 nm dual wavelength filter and images were captured using Q Capture-Pro 8.0 (UVP BioImaging Systems, Loughborough, U.K.).

### Cell cycle

The cell cycle distribution of leukemia cell lines was measured by the DNA contents in each cell based on flow cytometry using PI stain as described previously (Dahlawi et al. 2012). Data from 10,000 cells per sample were recorded and percentages of cells within G_0_/G_1_, S, and G_2_/M cell cycle phase were determined with FlowJo software and Waston (pragmatic) analysis of cell cycle (Tree Star).

### Liquid chromatography mass spectrometry

Samples (containing 20 μg gallic acid equivalents [GAE]s by Folin assay) were analyzed on a LCQ-DECA system, comprising Surveyor autosampler, pump and photo diode array detector (PDAD), and a Thermo-Finnegan mass spectrometer iontrap (Stoke on Trent, U.K.). The PDAD scanned three discrete channels at 280, 365, and 520 nm. Samples were eluted with a linear gradient of 5% acetonitrile (0.1% formic acid) to 30% acetonitrile (0.1% formic acid) on C18 column (Synergi Hydro C18 with polar end capping 2.0 mm × 150 mm) (Phenomenex, Hartsfield, U.K.) over 60 min at a rate of 200 μL/min. For the pellet samples, the gradient was altered (*t* = 0, 5% acetonitrile; *t* = 10 min, 12.5% acetonitrile; and *t* = 60 min, 30% acetonitrile) to try to separate the later eluting ellagitannins. The LCQ-DECA LC–MS was fitted with an electrospray ionization interface and the samples were analyzed in positive and negative ion mode. There were two scan events: full scan analysis followed by data-dependent MS/MS of the most intense ions. The data-dependant MS/MS used collision energies (source voltage) of 45% in wideband activation mode. The MS detector was tuned against cyanidin-3-*O*-glucoside (positive mode) and against ellagic acid (negative mode).

### Determination of total phenolics

The content of the total phenolics was evaluated by using the Half Strength Folin–Ciocalteu method adapted from (Deighton et al. 2000). First, 250 μL Folin–Ciocalteu reagent (Sigma Gillingham, U.K.) was added to 250 μL of each fraction (unbound, water, acetonitrile, acetone, and ethyl acetate) (1% diluted with water) and mixed gently prior to 3 min at room temperature. Then, 250 μL of sodium carbonate solution was added to each sample, mixed well, and incubated for 1 h prior to recording at 750 nm using Ultrospec™ 2100 *pro* UV/Visible Spectrophotometer (GE Healthcare Life Sciences, Buckinghamshire, U.K.). Gallic acid was used as a standard and total phenolic was expressed as GAEs.

### Pure compound treatments

Punicalagin and ellagic acid were purchased from Sigma (Pool, U.K.), and delphinidin-3-glucoside and cyanidin-3-glucoside were purchased from Extrasynthese (Geny, France). All four cell lines were treated with pure compounds at concentrations (0, 5, 10, 25, 50, and 100 μmol/L) for 24 h to test their effects on cell viability using the Cell Titer-Glo® Luminescent cell viability assay as described above.

### Statistical analysis

Means and standard error of the mean (SEM) were calculated and a Shapiro–Wilk test (Stats Direct, Cheshire, U.K.) was used to test whether data followed a normal distribution. Data were nonparametric and thus a Kruskal–Wallis test and a Conover–Inman post hoc test were used to investigate significant differences. Results were considered statistically significant when *P* ≤ 0.05.

## Results

### Differential effects of pomegranate fractions on ATP levels

**Figure 1:**
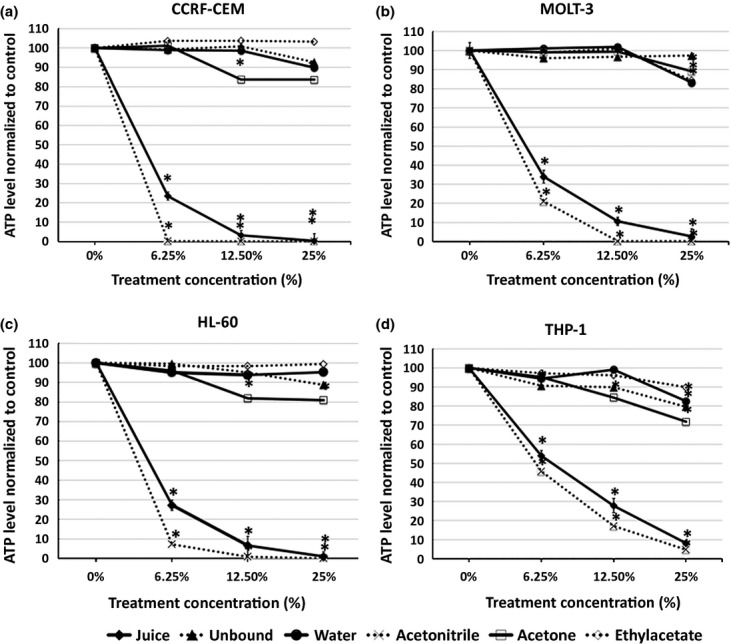
Effect of water, unbound, acetonitrile, acetone, and ethyl acetate fractions generated by SPE of PGJE together with whole PGJE in four leukemia cell lines (CCRF-CEM, MOLT-3, HL-60, and THP-1). Cells were treated for 48 h with all fractions at concentrations equivalent to the concentration of compounds within 6.25%, 12.5%, and 25% whole PGJE. ATP levels were investigated using the Cell Titer-Glo® Luminescent Cell Viability Assay to provide indication of live cell numbers. ATP levels normalized to controls and presented as means ± standard error. *Significant difference (*P* ≤ 0.05). SPE, solid phase extraction; PGJE, pomegranate juice extract; ATP, adenosine triphosphate.

Whole PGJE induced a dose-dependent inhibition of ATP levels within all four leukemia cell lines (*P* ≤ 0.05) (Fig. 1). The acetonitrile fraction obtained after SPE induced a significant decrease in ATP levels (*P* ≤ 0.05) which showed a nonsignificant increase in cytotoxicity compared to the whole juice extracts (Fig. 1). Low-dose treatments of leukemia cell lines with either whole PGJE or the acetonitrile fractions demonstrated dramatic reduction in ATP levels indicating decreased cell viability following 24 h treatment (Fig. 1). The unbound, water, acetone, and ethyl acetate SPE fractions had minimal effects on any cell line at the doses investigated (concentration equivalent to those present in 6.25%, 12.5%, and 25% whole PGJE treatments) (Fig. 1).

### Effect of acetonitrile fraction from pomegranate juice on cell cycle arrest within leukemia cell lines

**Figure 2:**
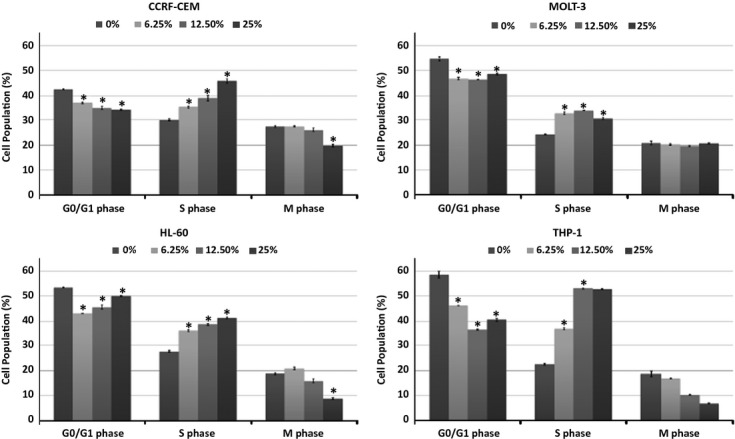
Effect of acetonitrile fraction generated by SPE of PGJE on cell cycle phase distribution in four leukemia cell lines (CCRF-CEM, MOLT-3, HL-60, and THP-1). Cells treated for 48 h with acetonitrile fraction at concentrations equivalent to those found in 6.25%, 12.5%, and 25% whole PGJEs. Means ± standard error shown. *Significant difference (*P* ≤ 0.05). SPE, solid phase extraction; PGJE, pomegranate juice extract.

Treatment of leukemia cells lines with the acetonitrile fraction for 48 h demonstrated significant S phase arrest within all four cell lines (*P* < 0.05) with a corresponding significant decrease in cells within G_0_/G_1_ phase at all concentrations (*P* < 0.05) (Fig. 2).

### Induction of apoptosis by acetonitrile SPE fraction from pomegranate extracts within leukemia cell lines

**Figure 3:**
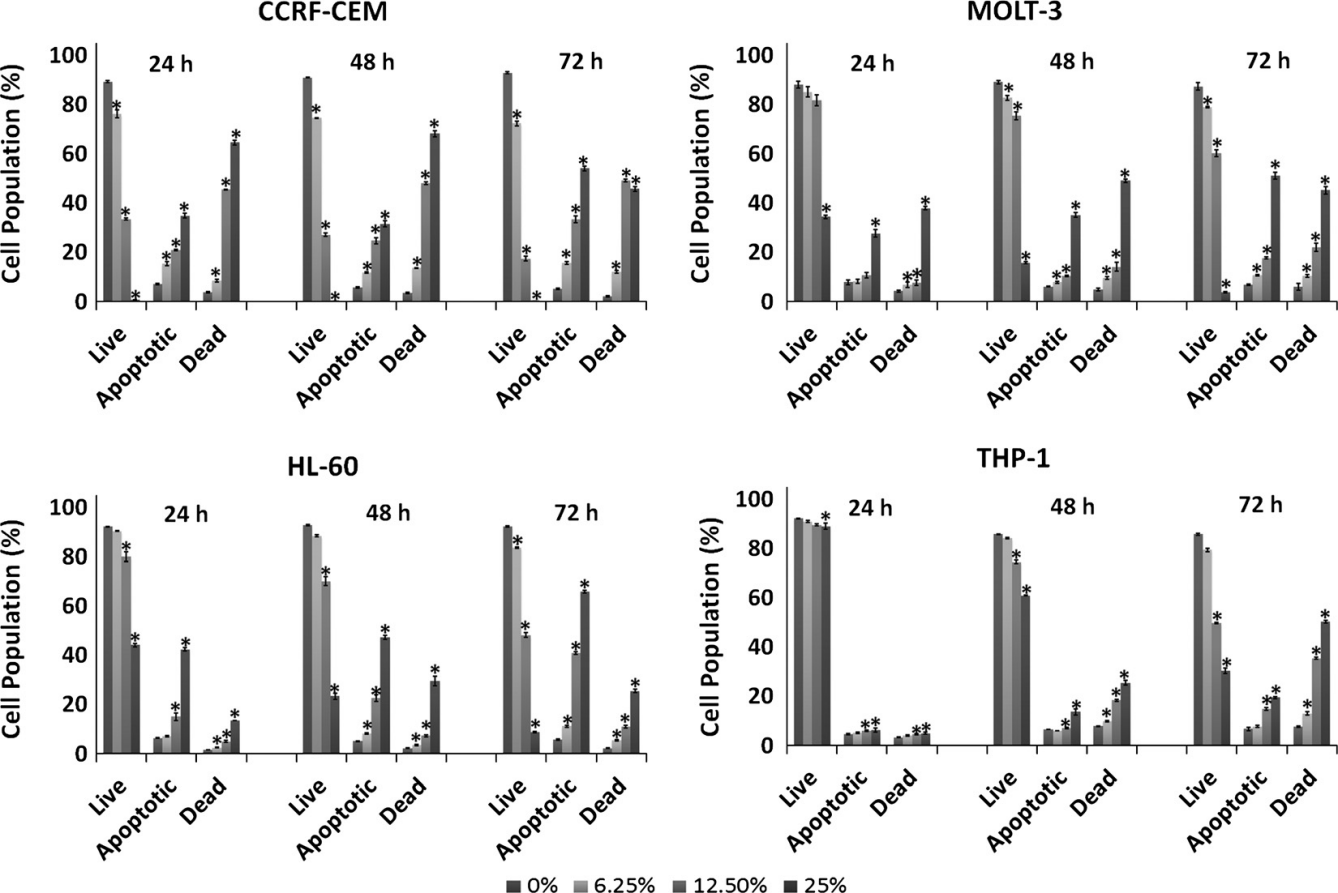
Effect of acetonitrile fraction generated by SPE of PGJE on induction of apoptosis in four leukemia cell lines (CCRF-CEM, MOLT-3, HL-60, and THP-1). Cells treated for 24, 48, and 72 h with acetonitrile fraction at concentration equivalent to the concentrations of compounds found in 6.25%, 12.5%, and 25% whole PGJE. Induction of apoptosis was determined by Annexin V-FITC/PI based on flow cytometry analysis. Means ± standard error. *Significant difference (*P* ≤ 0.05). SPE, solid phase extraction; PGJE, pomegranate juice extract; PI, propidium iodide.

**Figure 4:**
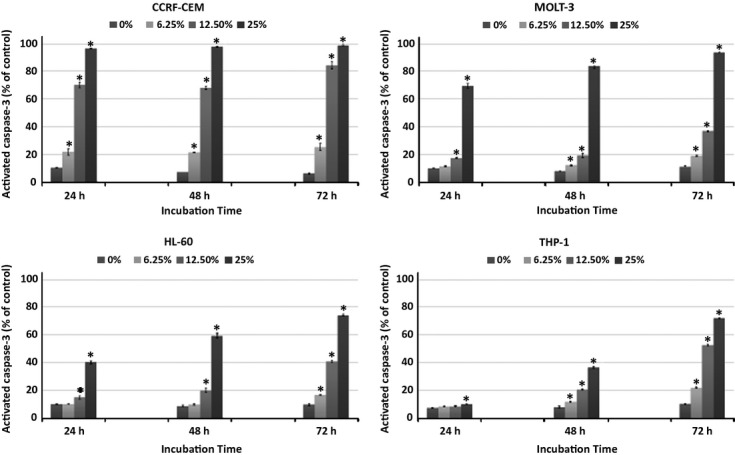
Effect of acetonitrile fraction generated by SPE of PGJE on caspase-3 activation in four leukemia cell lines (CCRF-CEM, MOLT-3, HL-60, and THP-1). Cells treated for 24, 48, and 72 h with acetonitrile fraction equivalent to the concentrations of compounds found in 6.25%, 12.5%, and 25% whole PGJE. Caspase-3 activation was determined by NucView™ 488 Caspase-3 substrate based on flow cytometry analysis. Means ± standard error of the mean. *Significant difference (*P* ≤ 0.05). SPE, solid phase extraction; PGJE, pomegranate juice extract.

**Figure 5:**
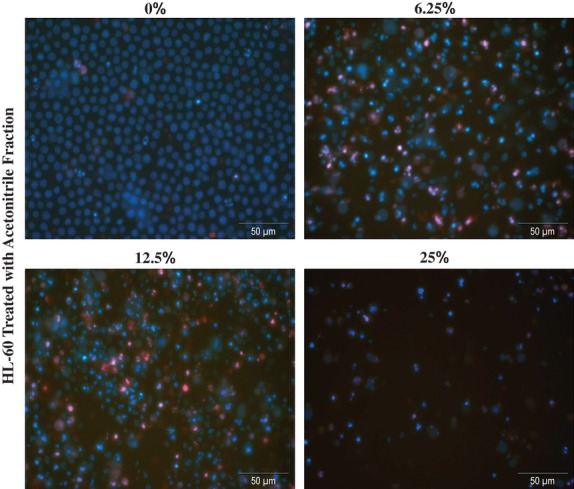
Effect of acetonitrile fraction generated by SPE of PGJE on morphology of apoptotic cells in HL-60. Cells treated for 48 h at concentration 6.25%, 12.5%, and 25%. Apoptotic morphology was determined by Hoechst 33258 and PI staining. SPE, solid phase extraction; PGJE, pomegranate juice extract; PI, propidium iodide.

The acetonitrile fraction induced apoptosis in a dose-dependent manner in all four leukemia cell lines following 24, 48, and 72 h incubation. CCRF-CEM was the most sensitive cell line and THP-1 cells were the least affected (Fig. 3). Induction of apoptosis by treatment with the acetonitrile fraction was confirmed with the induction of caspase-3 activity in a time- and dose-responsive manner in all cell lines (*P* < 0.05) (Fig. 4). Microscopic examination also demonstrated a marked increase in the number of cells displaying apoptotic morphology (such as chromatin condensation and fragmentation), in addition to a decrease in the number of live cells displaying normal nuclear morphology in all cell lines (Fig. 5).

### Phenolic content of SPE fractions

The acetonitrile fraction generated by SPE had the highest phenol content of 2696.7 μg GAE/mL, which was more than double than that seen within whole juice extracts (1236.9 μg GAE/mL), whereas the phenol content of other extracts was minor in comparison with 53.5 μg GAE/mL in the unbound fraction, 148.2 μg GAE/mL in the water fraction, 46.0 μg GAE/mL in the acetone fraction, and 5.7 μg GAE/mL in the ethyl acetate fraction. This translated to concentrations of phenol content of 6.25% equivalent treatments containing 5.2 μg/mL GAE within acetonitrile treatments and 2.4 μg/mL GAE within 6.25% treatments with whole PJE. Other fractions contained negligible levels of phenols which correlated with no observable biological activity (Fig. 1).

### LC–MS of whole PGJE and SPE Fractions

**Figure 6:**
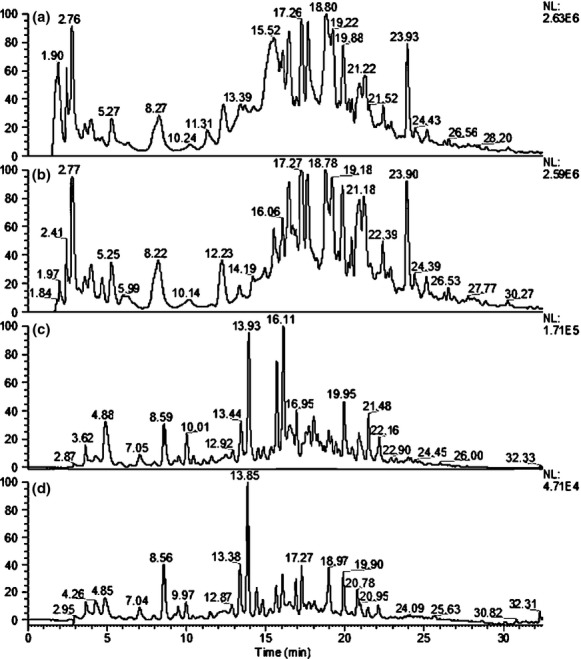
LC–MS chromatographs of whole PGJE (A) and acetonitrile (B), acetone (C) and ethyl acetate fractions (D) from solid phase extraction of PGJE LC–MS performed on samples containing 20 μg GAE/mL by follin assay. LC–MS, liquid chromatography mass spectrometry; PGJE, pomegranate juice extract; GAE, gallic acid equivalent.

**Figure 7:**
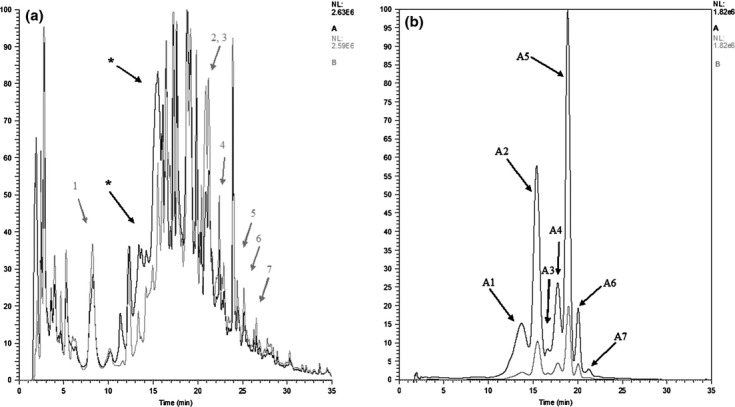
LC–MS chromatographs of whole PGJE (Black) and acetonitrile solid phase fraction from PGJE (Blue) demonstrating peak differences within negative ion mode (A) and positive ion mode (B). *The main peaks in juice sample that differ from the ACN fraction. The identities of peaks labeled are discussed in Table 1. LC–MS, liquid chromatography mass spectrometry; PGJE, pomegranate juice extract.

LC–MS was carried out on the whole PGJE and each fraction generated from the SPE procedure on samples containing 20 μg GAE. Virtually all the material from the unbound fraction and the water fraction was not retained and eluted in the column wash (data not shown). Previous work has shown that these fractions contain organic acids, vitamin C, and sugars. The juice contained peaks characteristic of phenolic components previously found in pomegranate (Fischer et al. 2011) and included gallotannins, ellagitannins, anthocyanins, and hydroxycinnamic acid derivatives (Figs. 6 and 7; Table 1). The acetonitrile fraction had the highest intensity of peaks but had a similar composition to the whole PGJE. The acetone and ethyl acetate fractions contained lower amounts of phenolic materials but certain components were enhanced in these fractions (Fig. 6). Comparison of whole PGJE and the acetonitrile fraction (Fig. 7A) demonstrated that the acetonitrile fraction was enriched in a number of ellagitannins, ellagic acid, and hydroxycinnamic acid derivatives over the juice (Fig. 7A and Table 1). However, it was clear that the acetonitrile fraction was depleted in anthocyanins compared to whole juice extract (Fig. 7A) but all the anthocyanins in the juice were also present in the acetonitrile fraction but at around eightfold lower levels (Fig. 7B; Table 1). The anthocyanins identified within both the whole juice extracts and acetonitrile fraction included the characteristic glycosylated forms of delphinidin, cyanidin, and pelargonidin (Fig. 7B; Table 1) found in pomegranate.

**Table 1:** Putative identification of phenolic components

Peak	PDA *max*	*m/z* [M-H]	MS^2^	Putative identity
1	275	783	481, 301	Punicalagin 1 (bis-HHDP-hexose)
2	275	1569	935, 785, 765	Sanguiin H-10-like isomer
3	275	1569	935, 785, 765	Sanguiin H-10-like isomer
4	280	1569	935, 785, 765	Sanguiin H-10-like isomer
5	275	581	301	EA derivative
6	290	507	327, 315	Methyl EA derivative
7	330	533	353	HCA derivative
A1	520	627^+^	303	Del-3, 5-diglc
A2	512	611^+^	287	Cy-3, 5-diglc
A3	500	595^+^	271	Pg-3, 5-diglc
A4	520	465^+^	303	Del-3-glc
A5	515	449^+^	287	Cy-3-glc
A6	500	433^+^	271	Pg-3-glc
A7	505	419^+^	289	Cy-pentose

Putative identifications are taken from (Mullen et al. 2002; Fischer et al. 2011). The MS data for the anthocyanins (peaks A1–7) was obtained in positive mode.

### Effect of pure compounds on ATP levels as an indicator of cell viability

**Figure 8:**
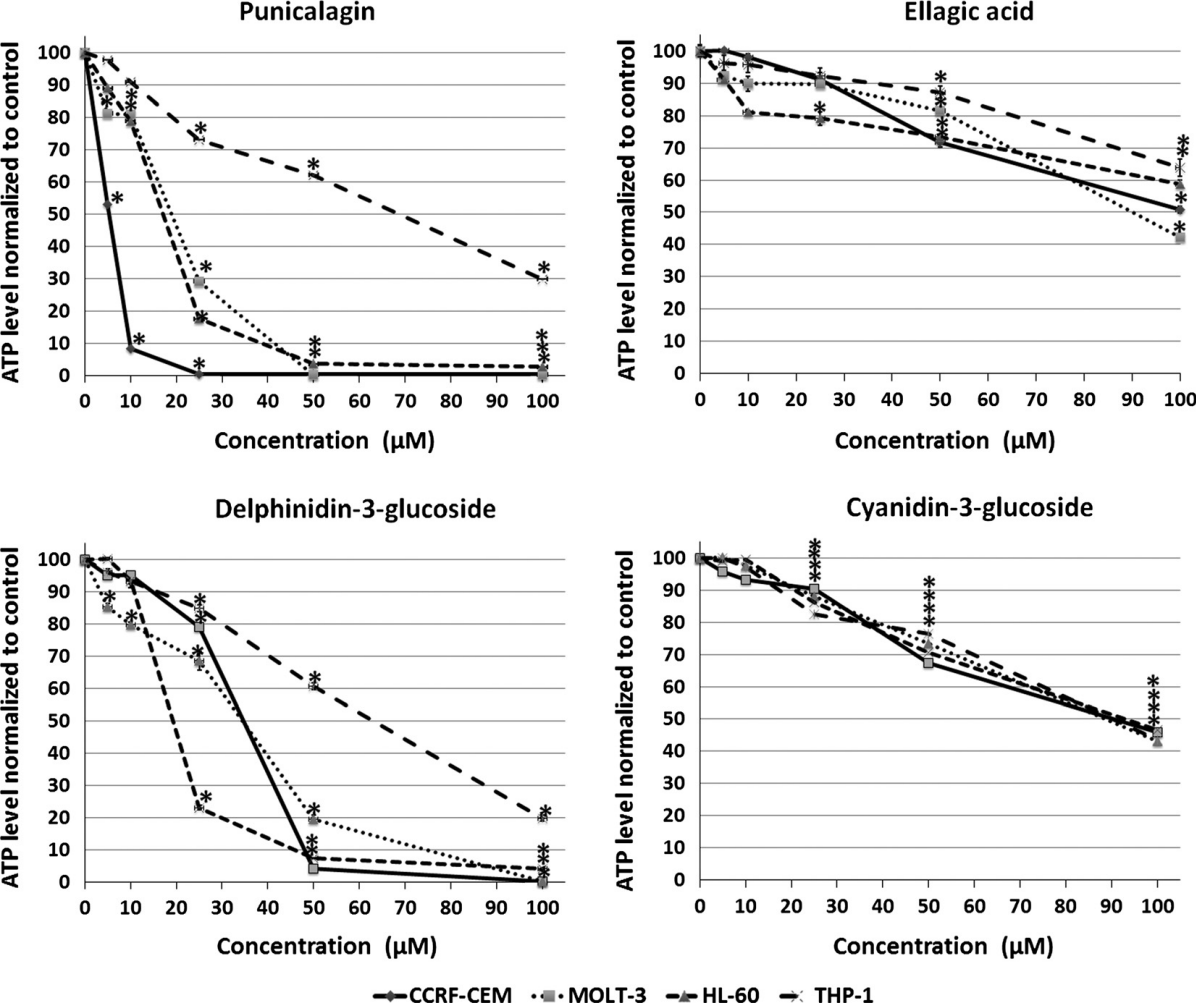
Effect of punicalagin, ellagic acid, delphinidin-3-glucoside, and cyanidin-3-glucoside on four leukemia cell lines (CCRF-CEM, MOLT-3, HL-60, and THP-1). Cells treated for 24 h with pure compounds at concentrations 0, 5, 10, 25, 50, and 100 μmol/L. ATP levels investigated using Cell Titer-Glo® Luminescent Cell Viability Assay to provide indication of live cell numbers. ATP levels normalized to controls and presented as means ± standard error. *Significant difference (*P* ≤ 0.05). ATP, adenosine triphosphate.

Pure compounds (punicalagin, ellagic acid, delphinidin-3-*O*-glucoside, and cyanidin-3-*O*-glucoside) significantly inhibited ATP levels in all four leukemia cell lines (Fig. 8). Punicalagin and delphinidin-3-*O*-glucoside were the most effective compounds although they displayed differential sensitivity toward the four cell lines investigated. Interestingly, the sensitivity of cell lines to punicalagin (IC_50_ CCRF [5 μmol/L] >HL-60 [17 μmol/L] >MOLT-3 [18 μmol/L] >THP-1 [69 μmol/L]) (Fig. 8) was identical to the order of sensitivity seen following acetonitrile fraction treatments (Fig. 1). Whilst delphinidin-3-glucoside was most toxic to HL-60 cells with an IC_50_ of 19 μmol/L compared to 35 μmol/L for CCRF and MOLT-3 (Fig. 8). Cyanidin-3-glucoside required high concentrations to inhibit 50% of cells (IC_50_: 89–91 μmol/L), whilst ellagic acid failed to reach 50% inhibition in all but MOLT-3 cells where a high dose of 89 μmol/L was required. Interestingly, THP-1 was the least sensitive to any compound a pattern mirrored with whole PGJ and that of the acetonitrile fraction.

## Discussion

Pomegranates have been postulated by a number of groups worldwide to provide bioactive compounds with anti-cancer actions, and have been successfully used in a number of randomized clinical trials for prostate cancer (Paller et al. 2012). We previously demonstrated that crude PGJ extract induced apoptosis and inhibited cellular proliferation inducing S phase arrest in leukemia cell lines (Dahlawi et al. 2012). Here, we used SPE to separate the PGJ into fractions and identified that the acetonitrile fraction contained the bioactive compounds responsible for induction of apoptosis, cell cycle arrest, and inhibition of proliferation within leukemia cell lines.

LC–MS analysis confirmed that the PGJE contained the mixture of gallotannins, ellagitannins, anthocyanins, and hydroxycinnamic acid derivatives characteristic of pomegranate (Fischer et al. 2011). Importantly, most of these components were recovered in the acetonitrile fraction with only a small proportion of phenolic components being identified in other fractions. The phenolic-enriched acetonitrile fraction was the most effective, and this strongly suggests that ellagitannins and other phenolic agents were responsible for its bioactive actions. Interestingly, we identified that the active acetonitrile fraction to be enriched in ellagitannins, but comparably depleted in anthocyanins.

A number of ellagitannins were identified as well as ellagic acid and hydroxycinnamic acid derivatives. Ellagitannins extracted from pomegranate have been previously shown to induce apoptosis in HL-60 cell lines (Li et al. 2011). We observed similar proapoptotic affects when we treated this cell line together with other leukemia cell lines with the ellagitannin-rich acetonitrile fraction from PGJ. Similarly, ellagic acid has been shown to induce apoptosis within HL-60 cell lines, together with S phase arrest (Hagiwara et al. 2010). This S phase arrest mimics that seen in our current study following treatment with the acetonitrile fraction and that seen in our previous study on whole PGJ (Dahlawi et al. 2012). In contrast, ellagic acid and caffeic acid treatment of normal human peripheral blood mononuclear cells inhibited apoptosis induced by hydrogen peroxide treatments, suggesting that these compounds have protective effects on these cells (Khanduja et al. 2006). This was confirmed in our previous study where PGJ extracts showed a greater toxicity toward leukemia cell lines than nontumor control cells (Dahlawi et al. 2012). This suggests that the ellagitannins within PGJ display protection to apoptosis in nontumor cells, but induce apoptosis in tumor cells. Treatment of cells with individual compounds demonstrated that ellagitannin: punicalagin mimicked the effects of the acetonitrile fraction from pomegranate suggesting that this may be one of the major bioactive compounds within pomegranate. Interestingly, in contrast, ellagic acid induced limited effects and failed to inhibit 50% of cellular ATP levels in the majority of cell lines, which combined with the inhibition of apoptosis seen previously by ellagic acid (Khanduja et al. 2006) suggests that this would not be useful in leukemia therapies.

Induction of apoptosis by a number of hydroxycinnamic acids have also been seen within leukemia cell lines. Artepillin C, an extract from Brazilian propolis, has been shown to induce apoptosis and inhibit DNA synthesis resulting in S phase arrest (Kimoto et al. 2001).

Anthocyanins were also found in the acetonitrile fraction from PGJ although at lower levels than seen in whole PGJ. Anthocyanin treatment of leukemia cell lines have also demonstrated induction of apoptosis by a number of groups worldwide (Hou et al. 2003, 2005; Katsuzaki et al. 2003; Fimognari et al. 2004, 2005; Hyun and Chung 2004; Chang et al. 2005; Xia et al. 2006; Feng et al. 2007; Lee et al. 2009). We too demonstrated that anthocyanin inhibited ATP levels suggesting affects on apoptosis. This could account for some of the apoptosis induction within leukemia cells following treatment with the acetonitrile fraction from PGJ. However, although treatment of U937 cells (histocytic leukemia cell lines) with cyanidin and malvidin demonstrated G_2_/M phase arrest (Hyun and Chung 2004), 50% effectiveness was not reached until ∼50 μg/mL. The acetonitrile fraction was very effective at ∼50 μg GAE/mL and the anthocyanin content was less than ∼5% of the content. The eightfold depletion in anthocyanin content between PGJE and the acetonitrile fraction did not reduce effectiveness and this suggests that the actions within PGJ may not be due to the anthocyanin content. This is further supported by the finding that neither anthocyanin investigated was as effective as punicalagin in any cell line.

This study demonstrated that only the acetonitrile fraction demonstrated activity toward leukemia cell lines suggesting that the phenolic agents within pomegranate are responsible for induction of apoptosis and inhibition of cell proliferation. The relative enrichment of ellagitannins over anthocyanins and the closer agreement on affects of cell cycle seen with ellagitannins treatment of leukemia cell lines, together with the lower IC_50_ concentrations seen for punicalagin compared to the anthocyanins, suggest that ellagitannins may be the major players in the anti-cancer actions of pomegranate. However, it is highly probable that a number of compounds found within the acetonitrile fraction of PGJ interact with each other. Indeed, synergistic actions of ellagic acid with quercetin and resveratrol have been demonstrated previously in the leukemia cell line MOLT 4 cells (Mertens-Talcott et al. 2003, 2005; Mertens-Talcott and Percival 2005). Seeram et al. (2005) found that individual treatment of human oral, colon, and prostate cancer cell lines with punicalagin, ellagic acid, and total pomegranate tannins failed to induce the same toxicity as whole pomegranate extracts suggesting that these agents either work synergistically or that other minor agents within PGJ are responsible for the actions of pomegranate (Seeram et al. 2004). Conversely, our study demonstrated that treatment of leukemia cell lines with the acetonitrile fraction from pomegranate at concentrations determined to ensure equal concentrations of agents (not just phenolics) within the fractions to those in whole PGJ suggesting all the toxicity seen in PGJ was due to the agents seen within this acetonitrile fraction. SPE resulted in increased phenolic content of the acetonitrile fraction compared to the whole juice, which was not surprising as it is concentrated in phenolic compounds due to the loss of sugars and other compounds in the unbound and water fractions. Further studies will focus on combination of treatments of agents found within this study to identify potential synergistic actions between agents.

Of course, upon consumption of pomegranates, many of the compounds identified here do not enter the blood stream within this form. For example, the ellagitannins themselves do not enter the bloodstream (Seeram et al. 2004, 2006, 2008; Espin et al. 2007). As such they cannot be effective directly against leukemic cells in vivo if delivered via oral consumption; however, these could be delivered via pharmacological means. However, oral delivery could still provide bioactivity from the ellagitannins as the colonic-derived metabolites, the urolithins, are known to circulate in the bloodstream (Seeram et al. 2004, 2006, 2008; Espin et al. 2007), accumulate at certain sites (Seeram et al. 2007), and are effective against cancer cell lines (Seeram et al. 2007; Bialonska et al. 2009; Kasimsetty et al. 2010), and thus could provide the bioactive actions of consumed pomegranates.

The identification of the compounds responsible within pomegranate for the anti-cancer effects and interactions between other natural compounds and chemotherapy agents will be the next step toward understanding the role of pomegranates in cancer therapeutics.
